# A qualitative exploration of information access and provision for cancer survivors experiencing chronic bowel symptoms after pelvic radiotherapy: informing a framework to guide improvements to information provision

**DOI:** 10.1007/s00520-026-11040-y

**Published:** 2026-09-22

**Authors:** Adam Biran, Christina Dobson, Jack Maybury, Colin J. Rees, Rachel Brooks-Pearson, Anthony Cunliffe, Lisa Durrant, John Hancock, Laura Jane Neilson, Ana Wilson, Linda Sharp

**Affiliations:** 1 Population Health Sciences Institute, Newcastle University, Newcastle-Upon-Tyne, UK; 2 NIHR Newcastle Patient Safety Research Collaboration (PSRC), Newcastle Upon Tyne, UK; 3 Northern School of Radiology, Newcastle Upon Tyne, UK; 4 Northern Centre for Cancer Care), Newcastle Upon Tyne Hospitals NHS Foundation Trust, Newcastle Upon Tyne, UK; 5 Newcastle University, (Translational and Clinical Research Institute), Newcastle Upon Tyne, UK; 6 South East London Cancer Alliance, London, UK; 7 Somerset NHS Foundation Trust, Taunton, UK; 8 Faculty of Health and Life Sciences, University of Exeter, Exeter, UK; 9 North Tees and Hartlepool NHS Foundation Trust, Hartlepool, UK; 10 Department of Gastroenterology, South Tyneside and Sunderland NHS Foundation Trust, South Shields, UK; 11 Imperial College London, London, UK; 12 St Mark’s Hospital, Harrow, London, UK

**Keywords:** Cancer, Pelvic radiation disease, Information, Qualitative study, Radiotherapy late-effects

## Abstract

**Purpose:**

For people living with and beyond cancer, access to clear, accurate, relevant information is crucial for meaningful participation in shared decision-making and is associated with better quality-of-life. Many cancer patients who undergo pelvic radiation experience chronic bowel symptoms, decreasing quality-of-life. We aimed to explore information access among survivors with experience of chronic bowel symptoms and develop a framework to guide future information provision.

**Methods:**

We conducted cross-sectional, semi-structured interviews with 28 cancer survivors (14 prostate, 10 gynecological, 4 anal/rectal) with experience of chronic bowel symptoms after pelvic radiotherapy, and 19 health professionals who provide treatment and care for survivors with these symptoms. Participants were recruited through UK cancer charities and National Health Service hospitals. Data were analyzed thematically. Findings informed a framework to guide improvements to information provision, developed with a patient panel.

**Results:**

Data were organized to reflect survivor experiences with information access and provision at different points along their cancer journey: *Pre-treatment; Recognizing Symptoms; Managing* S*ymptoms;* and two cross-cutting categories of *Information Sources* and *Challenges in Information Provision.* The framework encompasses information purpose and channels, survivors’ information needs at different time points and challenges and considerations for implementation.

**Conclusion:**

Access to timely, relevant, accurate information was an issue across the cancer pathway for this survivor group. Participants reported disparate information needs which could not have been fully known at treatment outset, and which changed over time. Our framework offers a potential starting point for efforts to improve information access and provision.

**Supplementary information:**

The online version contains supplementary material available at https://doi.org/10.1007/s00520-026-11040-y.

## Introduction

Access to appropriate, clear, and relevant information after cancer diagnosis allows meaningful participation in treatment decisions and is associated with lower depression and anxiety and better quality-of-life among cancer survivors (see, for example, [1, 2]). Access to high-quality information can also support lifestyle changes to prepare for treatment and, potentially, reduce risk of recurrence [3]. However, many survivors are not satisfied with the information they access or receive [4]. Information needs are among the most frequently reported unmet supportive-care needs among people affected by cancer [5]. Survivors commonly express unmet information needs related to symptoms and treatment, lifestyle changes after cancer, and follow-up and testing [6, 7].

Information needs can differ by cancer site [8] and change over time as individuals move through diagnosis, treatment, recovery, and survivorship [9]. Previous studies have considered information needs associated with specific cancers including breast, prostate, and colorectal [1, 2, 10]. However, other groups of survivors who share a common experience, such as undergoing a particular cancer treatment, may also have specific information needs. One such group is those sharing the common experience of chronic bowel symptoms following pelvic radiotherapy. Typical symptoms include diarrhea and constipation, urgency, and loss of control over bowel movements, some of which are common [11], and some of which can be severely debilitating and impact many aspects of people’s lives [12].

Supported self-management is increasingly seen as central to the long-term care of people with chronic conditions including cancer [13–15]. Self-management support can improve patient-reported outcomes and reduce healthcare costs [16]. As regards those with post-radiotherapy bowel symptoms, to be able to effectively self-manage, they must be both supported and recognize that the symptoms they are experiencing are the result of radiotherapy. In terms of the latter, emerging symptoms may not be attributed to radiotherapy because (i) they are unexpected; (ii) they often develop months or years after radiotherapy completion, and (iii) their nature (e.g. often multiple and disparate) means they can easily be misattributed to other causes [17, 18]. After symptoms are appraised as a potential consequence of treatment, many people face challenges raising these with medical professionals, sometimes because of feelings of embarrassment, or reluctance to be perceived as complaining, when they should “just be grateful to be alive” [18]. Timely access to appropriate information may be particularly important for this group to both increase their knowledge and empower them to seek help and/or self-manage. However, little is currently known about patient experiences of information provision, and unmet needs, and access in relation to chronic bowel symptoms post-radiotherapy.

In this exploratory, descriptive study of people experiencing chronic bowel symptoms following pelvic radiotherapy, our primary objective was to report, in participants’ own words, their experiences with information access and provision and the implications for experiences with bowel symptoms and their management. A secondary objective was to suggest a framework to systematically consider the information needs of this survivor group, serving as a potential starting point for efforts to improve information provision, and consequently, improved self-management.

## Methods

Methods are presented in detail elsewhere [12, 18] (https://tinyurl.com/4y3t5t43, https://tinyurl.com/3sa595ed) and are summarized below.

### Design

This was a cross-sectional qualitative study.

### Sampling and recruitment

Two participant groups were recruited: cancer survivors with experience of bowel symptoms after pelvic radiotherapy and health professionals involved in the care of cancer survivors with chronic bowel symptoms. Participants were recruited through UK cancer charities, professional networks, and five participating National Health Service (NHS) Hospital Trusts. Following Dworkin [19], we anticipated a sample size of around twenty individuals per group would allow us to reach a point where further interviews would add little by way of new information.

### Data collection

Data were collected through semi-structured interviews between November 2021 and September 2022. AB (a trained, experienced, male, qualitative researcher, with no medical training, no personal experience of cancer, and no prior relationship or contact with participants) conducted all interviews. Interviews were conducted remotely (by phone or online using Microsoft Teams) and lasted up to 90 min for survivors or 60 min for health professionals. Interviews flexibly followed a topic guide (Supplementary File 1), one for survivors, another for health professionals. Participating survivors (but not health professionals) were offered a £25 voucher for participating.

### Analysis

Interviews were digitally recorded, transcribed and pseudonymised. After familiarisation, transcripts were imported into NVivo software and coded by AB and CD. The initial coding framework, which reflected the topic guides, was applied to six transcripts (three survivor and three health professional) independently by AB and CD to check the usefulness, credibility and comprehensiveness of the coding scheme. Additional codes were identified across transcripts and incorporated into a revised coding framework which was applied to the remainder of the transcripts. When further codes were identified these were incorporated into the framework and previously coded transcripts reviewed to look for any instances of these. Coded data were analysed thematically [20] by AB with a focus, in this analysis, on participants’ accounts of information seeking, access and provision. The analysis included the relevant data from the survivors and health professionals. We adopted a subtle realist paradigm, assuming that participants’ accounts provide a meaningful reflection of their experiences, while acknowledging the potential influence of context and perspective. We report findings using participants’ own words, presenting these at a largely descriptive level to support service improvement.

Informed by the experiences described by our participants, we developed a framework to inform consideration of information provision, content, and timing. This was reviewed by five public and patient representatives with personal experience of bowel symptoms post-cancer (who had not taken part in interviews) and revised accordingly.

### Consent and ethical review

The study was conducted in accordance with recognized ethical standards for health research in the UK and in line with the principles of the Declaration of Helsinki. All participants provided informed consent verbally, responding explicitly to each of a scripted set of seven statements read out by the interviewer immediately prior to interview. This was audio recorded. The study received a favorable opinion from the Health and Care Research Wales, Research Ethics Committee on 12th August 2021 (Reference 21/PR/1138).

The study is reported following the Standards for Reporting Qualitative Research (SRQR).

## Findings

### Sample characteristics

Our sample comprised 28 cancer survivors and 19 health professionals. Further details are shown in Tables 1 and 2.

**Table 1:** Sample size by gender (survivors) and professional role (health professionals)

Participant group and gender / role	Number of participants
*Survivors*	
Female	14
Male	14
*Health professionals*	
Gastroenterology consultant	4
Oncology consultant	4
Therapeutic radiographer	2
Clinical nurse endoscopist	2
Clinical nurse manager	1
Physiotherapist	1
Dietician	1
Late-effects service lead	1
Endoscopist	1
GP	1
Consultant colorectal surgeon	1

**Table 2:** Sample characteristics (survivors)

Sample characteristic	No. participants	
	**Female (n = 14)**	**Male (n = 14)**
Recruitment route		
*Charity*	11	2
*NHS Trust*	3	12
Age in years, median (range)	59.5 (34–75)	70 (64–82)
Cancer site		
*Prostate*	-	14
*Cervix*	5	-
*Endometrium/uterus*	5	-
*Anus/rectum*	4	-
Years since pelvic radiotherapy, median (range)	5 (1–17)	3.5 (0.5–7)
Ethnicity		
*White*	14	13
*Black*	-	1
Bowel symptoms self-reported at recruitment		
*Bowel frequency/urgency*	10	
*Bleeding*	2	10
*Pain*	10	3
*Diarrhoea*	6	4
*Excess wind*	8	5
*Unable to empty bowel*	8	4
*Tenesmus*	5	4

We organised data into three temporal categories, to bring together survivors’ experiences with information access and provision at different points along their cancer journey *Pre-treatment*, *Recognising Symptoms* and *Managing* S*ymptoms*. This reflected a narrative structure that was common in survivors’ accounts and was expected to provide an accessible organising structure for our information needs framework. Additionally, two cross-cutting categories, *Information Seeking & Sources* and *Challenges in Information Provision* were used for data which described features of, and issues with, information access and provision across the timeline. We outline these categories below with illustrative quotes from survivors to foreground their experiences (Supplementary Table 1 includes additional quotes from survivors and professionals).

### Pre-treatment

Participants discussed three main topics in relation to information received pre-treatment, namely information: *Provision*; *Reception*; and *Use*.

#### Provision

Before treatment, health professionals reported making patients aware of possible short and long-term effects of treatment, to facilitate informed decision-making about treatment and for consent processes. Some survivors recalled this information being given, sometimes as printed material; some were satisfied with the information provided while others considered it inadequate. Not all recalled receiving information about possible late effects.

The hospital gave me information at the start, but it wasn’t highlighted…that there could be an ongoing problem caused by the radiotherapy. (ID103, male, 66 years)

#### Reception

Before treatment, survivors’ attention often focussed on the diagnosis and immediate treatment. Participants’ priority was survival; treatment was perceived as essential for this and everything else secondary, making it difficult for them to take in details about possible late-effects.

I just wanted to, you know, not die. So, it went in, but it was kind of at the back of my mind, it was more about the then and now (ID110, female, 48 years)

#### Use

Information access prior to treatment was intended to allow informed choice about, and consent for, treatment. One prostate cancer survivor described researching treatment options prior to making a choice, and a gynecological cancer survivor described difficulty getting the information she believed would have allowed her to make a more informed decision. However, most perceived no meaningful choice to be made about the radiotherapy, with it ultimately being necessary for survival.

I couldn’t make an informed decision because there was no decision to make, I had to have it. (ID110, female, 48 years)

### Recognising symptoms

Participants reported how information provision impacted their experiences with radiotherapy late effects. Some described being unprepared for late effects such as incontinence and sometimes were alarmed by them. One health professional noted that survivors could find the experience of anal bleeding particularly alarming if they were unprepared for it, and similarly one survivor described how bleeding was not alarming once the cause was understood. Several survivors noted that they would have liked to be better informed about these symptoms in advance. Some described an experience of sudden realization when they came across information that described or explained their symptoms.

I was just scrolling a news feed and this PRDA (Pelvic Radiation Disease Association) popped up, and I thought I’ve never heard of pelvic radiation damage, so I read it and it’s just like a light bulb, and I thought my God that’s me… (ID111, female, 65 years).

### Managing symptoms

Some survivors sought information to help manage their symptoms. This was primarily described in relation to diet (as they suspected that their symptoms were influenced by what they ate and drank), though one survivor mentioned seeking information regarding exercise and another recalled getting advice about incontinence pads. Information could be hard to find, and survivors reported attempts to figure things out for themselves in the absence of any guidance, with some potentially imposing unnecessary self-restrictions and others wondering whether there were any further changes they should try. Some health professionals also recognized the importance of dietary advice and support to help people avoid over-restricting their food intake and to find appropriate alternatives.

They put me in touch with the dietician in the [hospital], and honestly when I explained what was going on and the treatment, it was woah I’m sorry but there’s nothing I can do for you absolutely nothing I can do for you. …So, I just did my own (diet). (ID101, female, 66 years).

### Information seeking and sources

Survivors differed in their information wants, with some actively seeking it and others preferring to avoid information.

It’s a good idea to get as much information about this sort of thing as you can (ID129, male 67 years)

I tend not to look things up medically because you can frighten yourself (ID111, female, 65 years)

In addition to the information received from health professionals, usually prior to treatment (see above), participants commonly mentioned cancer charities and the NHS generally as sources of information and, less often, academic literature, support groups, and word-of-mouth. Except for word-of-mouth, these were frequently, though not exclusively, accessed via the internet, which was also used for wider information searches. Participants recognized the possibility of accessing misinformation, and the NHS, cancer charities, and academic literature were cited as trusted sources.

if you’re new to it the information you need to find is gonna be correct is the NHS site, MacMillan or Jo’s Trust. They’re the three kind of places that I know have got the most up to date proper information (ID110, female, 48 years).

Several participants mentioned participating in support groups, using these to look for specific information as well as to learn about others’ symptoms and experiences, sometimes as a point of comparison. However, not all survivors wanted to participate in these groups, and one also noted that groups could be a source of misinformation.

### Challenges in information provision

Our data suggested multiple challenges for, and gaps in, information provision. We organised participants’ experiences relating to these around three topics, *Timing*, *Format* and *Content*.

#### Timing

Survivors and health professionals recognized that prior to treatment, patients may not be receptive to information about possible late effects, and some suggested that the end of treatment may be an important time to provide this information.

I think when you’re coming to the end of your radiation, we should be told, you know…that there is a chance that, you can have longer term effects and here’s some more information on it and here’s what to look out for. (ID108, female, 34 years).

However, it was also recognised that even information given at the end of treatment may not be retained to the point at which symptoms appear, and one health professional noted an issue of ‘legacy patients’, who were treated a long time ago and are now hard to reach with information. In view of these challenges, one health professional highlighted the potential importance of a process of checking and repeating information rather than one-off provision or access.

#### Format

Survivors and health professionals described how the time prior to treatment was when a lot of information was imparted. When this was done verbally, it could be affected by the limited health professional time available. However, printed materials had a mixed reception, with some survivors commenting on the volume of material provided being difficult to assimilate.

…it was a great heap of paper about this, that and the other and you flicked through it (ID118, male, 78 years)

#### Content

Some participants stated they believed that information provision for survivors had improved since their experience. However, gaps in current provision were also noted. One survivor suggested it would be useful to have information on how to discuss late-effects symptoms and support needs with employers and pointed to a gap in provision of information about late-effects for young adults who have left home but not yet started a family of their own. Another highlighted the potential importance of learning about personal experiences of living with and managing symptoms in addition to medical information. Two survivors talked about information for health professionals, one noting that this information is important to enable professionals to provide effective support for patients with different needs, the other noting that although information had improved for health professionals as well as patients since her diagnosis, it seemed slow to permeate through to practitioners.

…for me, it lies more with the professionals that you’re dealing with because not everyone will have side effects and not everyone’s will be the same, but your professionals that you’re dealing with are your team and they should know your individual case and be able to help you, (ID108, female, 34 years).

### Framework for information provision

Supplementary Table 2 shows the proposed information provision framework. It considers likely information needs at different time points in the experience of cancer, the purpose of the information, the sources or contact points by which information might be passed between health services and survivors and some of the issues that might be associated with this.

## Discussion

While some survivors living with chronic bowel symptoms after pelvic radiotherapy were satisfied with their information access, others struggled to get the information they wanted at the time they wanted it. We found unsatisfactory access to information led participants to report problems with informed decision-making and being unprepared for late-effects when they arose. The period before treatment was a common timepoint for information provision, but the volume of information could be overwhelming, and was not always accessed or retained if not pertinent to immediate needs. For some survivors, alarm at the onset of bowel symptoms was reduced once they better understood their symptoms’ cause. Information was also important to assist self-management if chronic symptoms were experienced. Individual information needs varied by symptom experience, life stage and preferences and could change as the symptom and treatment context progressed.

There are several contributions of this paper. Firstly, it adds to the limited qualitative literature exploring the lived experience of those who have had chronic bowel symptoms following pelvic radiotherapy. This is a growing population [21], many of whom struggle with self-management of symptoms, leading to profound quality-of-life impacts [12]. Furthermore, our participant group included survivors of gynecological cancers, an underrepresented population in a literature dominated by prostate cancer studies [11]. Participants had experienced various pelvic cancers, were 0.5–17 years post-treatment and were able to reflect on experiences across their journey. Secondly, it extends this limited evidence-base to explicitly consider the information needs of this group. Thirdly, based on our findings, we developed a novel provisional framework for information access; this framework can now be used to inform the future design and development of information resources to support patient-centred care and self-management for cancer survivors treated with pelvic radiation.

Our findings highlight the importance of information access and provision for supportive care in this survivor group, and participants showed a variety of information needs. Following Rutten [9] these could be classified as treatment-related information (risks and benefits of treatment), rehabilitation information (long-term side-effects of treatment), coping information (support from groups and other patients) and social information (effects on work life). Individual participants varied in their information experiences with some clearly satisfied and others not. Individual differences in needs have been noted before and may reflect differences in individuals’ symptom experiences [22]. Bowel symptoms experienced by survivors following pelvic radiotherapy show considerable variation in nature and severity as well as in timing of onset relative to treatment [11, 18] and our participants exhibited similar variation [12].

Several participants mentioned the value of hearing personal experiences from other survivors, accessed through support groups or chance encounters, both as a source of advice on self-care and to inform choices about treatment. Some also described using support groups to place their symptom experiences relative to those of other survivors. Previous work has noted the value of learning of personal experiences from other survivors [22], however, the possibility of misinformation through these channels was raised by participants as a risk. Another risk, apparent from some participants’ accounts, is that realising other people have more severe symptoms could increase a sense of gratitude and acceptance of symptoms. Our earlier paper, using data from this same participant group, suggests that gratitude and acceptance of symptoms can inhibit care-seeking, leaving survivors to continue unsupported [18]. Similar findings among colorectal cancer survivors, post-surgery were reported in a qualitative study [23], suggesting this is a not uncommon risk. A third risk is that undue weight might be given in treatment decision-making by patients to salient personal experiences over a more rigorous body of evidence e.g. [24]. While we have no evidence for this leading to poor decision-making in our participant group, one participant described the importance attached to the personal experiences of other known cancer survivors in making his choice of treatment, highlighting at least the potentially powerful influence of information from this type of source. This may be particularly pertinent at the current time with the rapid growth in the spread of medical misinformation.

The timing of information provision was an issue for health professionals in our study as well as survivors. Information provided prior to treatment could inform treatment decisions, if patients believed there were viable options for choice. However, patients may not be receptive to information on late effects while treatment itself is ongoing or yet to start (e.g. [4]). While information provided at the end of treatment might leave survivors better prepared if late effects arise soon after treatment, for some survivors symptoms do not appear until years after treatment. In view of this, the challenge is how to ensure survivors are sufficiently aware of the possibility of symptoms and provide a source that they will return to for advice should symptoms arise.

Several participants mentioned seeking information on diet modification for symptom control, and professionals noted that they had experience of some survivors unnecessarily restricting their diets. Survivors often failed to find the information and support they sought, sometimes notwithstanding having contact with a dietitian, and described trying to figure things out for themselves based on trial and error. Similar findings were reported from a study of colorectal cancer survivors post-surgery [23]. For our participants, provision of effective generic advice may be complicated by the existence of different possible causal mechanisms producing identical symptoms but potentially with different implications for dietary intervention.

Our findings suggest that a significant challenge in information provision for the management of bowel symptoms will be how best to serve a survivor group whose disparate needs cannot be known or predicted at the end of their active cancer treatment. With the move away from hospital-based follow-up care towards supported self-management in many healthcare systems (e.g. [15]), if symptoms arise, appropriate support and effective self-management will likely require symptom recognition and care-seeking on the part of survivors, as well as input from well-informed health professionals. The probability of this occurring may be increased if patients are provided with information on possible symptoms and appropriate courses of action at the point of discharge. However, given that symptoms may arise years or even decades after treatment, this information may not be retained when symptoms are experienced. It may therefore be necessary to ensure this information remains accessible through other routes, such as trusted web sources (e.g. cancer charities and the NHS) and perhaps through routine contact with health services. Moreover, as we have previously suggested, to encourage care-seeking, information may need to help survivors overcome strong social norms associated with gratitude (for successful treatment of cancer) and disgust and embarrassment (around discussion of bowel symptoms) [18].

The provisional framework we propose is intended as a starting point for thinking about information needs and challenges of provision at different timepoints relative to a cancer diagnosis and with a perspective informed by survivors’ experiences. It is intended to provide a foundation for supporting self-management for this patient group, and to complement other initiatives designed to improve care experiences and outcomes of cancer survivors, such as survivorship care plans, electronic self-reporting of symptoms, and nurse-led survivorship clinics [25–27]. It is evident from our results that some barriers exist at the patient level while others lie within the healthcare system. The framework, therefore, combines patient-level (e.g. layering information for more personalised access and better retention) with systems-level (e.g. the potential roles of primary and secondary care and associated support services) considerations and, importantly, highlights information needs around re-entry to the health service and the associated barriers for under-recognised symptoms with a delayed onset. Many options for implementation are available across different, or multiple, channels of communication, to address barriers at different levels: for example, at system/professional level, a clinical checklist and/or integration within existing clinical record systems, or at patient-level, a web-app and/or clinically endorsed videos of lived experience incorporating reliable and accessible information. Solutions might vary locally depending on existing systems and standard practice as well as available resources. Therefore, next steps should involve refining and optimising the framework in collaboration with both survivors and practitioners, followed by iterative co-development of resources to improve information provision.

Finally, although developed for those experiencing post-radiotherapy bowel problems, it is likely that the elements of this framework will apply more generally across survivors who share common experiences of other forms of cancer treatment (e.g. endocrine therapy, immunotherapy), with some tailoring for the specific types (and timing) of symptoms experienced.

### Limitations

All our participants but one self-identified as White British. We did not include people treated with pelvic radiotherapy who had no subsequent experience of chronic bowel symptoms. This group would include survivors experiencing other symptoms of pelvic radiation disease as well as those who were symptom-free. The information needs of this group may be different. Furthermore, while our participants were able to reflect on their experiences at different time points along the treatment pathway, memories of these experiences may have changed over time, and memories of earlier experiences may be colored by subsequent events. Recruitment through cancer charities may have resulted in a sample skewed toward more active and engaged individuals who may be more likely to have identified unmet information needs and to have sought information. Future research should explore the information needs and challenges of patients with symptoms who have not engaged with charities; this might involve community-based recruitment approaches, particularly for marginalized groups or survivors with lower health literacy, who may find it more difficult to access health information. Additionally, our participants could only report on information needs relating to support of which they were aware or had experienced. There may also be other support services, such as specialist physiotherapy, not mentioned by our participants but potentially beneficial and about which information might usefully be disseminated. Finally, information was only one topic covered in the interviews, and greater depth and detail may have resulted from interviews focused solely on this.

## Conclusions

Access to timely, relevant, and accurate information was an issue across the cancer journey for this patient group. Individuals have disparate information needs which cannot be fully known at the outset, and which change over the course of their cancer journey. Our framework offers a potential starting point for efforts to improve information access and provision. A critical next step will be to iterate this framework, based upon further feedback from survivors, particularly those from marginalized groups not represented in our study population, who may face different or additional information needs and challenges, as well as health professionals, to inform development of potential materials for future information provision.

## Supplementary information

Below is the link to the electronic supplementary material.

Supplementary file1 (DOCX 38 kb)

Supplementary file2 (DOCX 34 kb)

Supplementary file3 (DOCX 19 kb)

## Data Availability

The data supporting the findings of this study are held securely on Newcastle University servers and will be retained for 10 years following the conclusion of the study in accordance with institutional data governance policies. Anonymised data can be made available to researchers upon reasonable request to the corresponding author, subject to appropriate ethical and data protection considerations.
