# Supplementary material for: A qualitative exploration of information access and provision for cancer survivors experiencing chronic bowel symptoms after pelvic radiotherapy: informing a framework to guide improvements to information provision
Source: Support Care Cancer. 2026 Sep 22;34(10):999. doi: 10.1007/s00520-026-11040-y (PMC13597550; doi:10.1007/s00520-026-11040-y)
Supplement: Supplementary file 1 [file 520_2026_11040_MOESM1_ESM.docx]

**Survivors/patients interview topic guide:**

This guide is to general topics that will covered in the interviews. It will be used flexibly, so that the participant can share information in a way that is most natural to them. How questions are asked and the order in which they will be asked will vary from interview to interview. If a new topic of interest arises in one interview, this may be added to the guide for inclusion in subsequent interviews.

*Throughout, be alert to and probe for possible different routes to support and different types of support – who do they go to and what are they looking for?*

**Topic and sub-topics**

A) **History**
I. Brief history of diagnosis and treatment

B) **Symptoms**
I. Current symptoms
II. History of symptoms – including during radiotherapy (if any) and how they have evolved over time
III. What did you think these symptoms were
IV. Expectations of symptoms – initial occurrence and future expectations; information provided before/after radiotherapy
V. How do you feel about these symptoms now? Why? Has this changed over time? If yes, why?

C) **How symptoms impact on daily life**
I. **Activities** - work – leisure – domestic – family/relationships – diet – travel – anything else
II. **Psychological** – anxiety – depression - mood
III. **Recovery** – return to normalcy

D) **Help and support needs and experience**
I. From within health system & from outside the health system (e.g. online support)
II. History of support-seeking, type of support sought, experience of support given (prompts: cancer team; GP; ever offered referral; informal sources of support)
III. Why was treatment/support sought through chosen pathway(s); why was no treatment/support sought (if that applies)
IV. Satisfaction with interactions and their outcomes

E) **Self-management strategies**
I. Is there anything you do, or anything you have tried yourself, to manage your symptoms and the way they affect your life?
   - What, when, why, with what effect?

**Health Professionals Topic Guide**

This guide is to general topics that will covered in the interviews. It will be used flexibly, so that the participant has the opportunity to share information in a way that is most natural to them. How questions are asked and the order in which they will be asked will vary from interview to interview. If a new topic of interest arises in one interview, this may be added to the guide for inclusion in subsequent interviews.

**Topic and sub-topics**

**A) Contact with patients, post-radiotherapy**

(I) Introductory questions – on experience with patients, post-radiotherapy – before moving to symptoms
(II) How commonly do you see patients reporting bowel symptoms following pelvic radiotherapy?
(III) What is/are the route(s) by which they usually come to you?

**B) Symptoms and patient expectations**

I) What symptoms do they usually present with?
II) Which symptoms are the ones they find most distressing or bothersome? – Why?
III) What are their expectations / desires/ needs in terms of treatment or support?

**C) Treatment / support options and choices**

I) What options are available for you to offer in terms of treatment or support?
II) What would you usually offer in terms of treatment or support?
III) Why / how do you make this choice?
IV) Is there any guidance or protocol which you follow?
V) How useful/ helpful do you find this guidance? / Do you think there is a need for more or better guidance/ guidelines? Why?

**D) Effectiveness of treatment options**

I) How effective do you think the treatment/ support that you are able to offer is?
II) Has your approach to this changed over time – How? Why?
III) How satisfied are patients with what you can do for them? Why?
IV) What are your expectations for these patients following treatment?

**D) Gaps in provision – (specific to respondent)**

I) Where do you see the most significant gaps in the treatment/support you are able to offer?
II) Is there a group of patients for whom you are unable to offer any effective treatment / support?
III) What characterises this group?

**E) Gaps in provision – (across health service)**

I) To what extent do you think the health service meets the needs of patients with symptoms consistent with Pelvic Radiation Disease (PRD)? – Why?
II) What do you think would constitute a worthwhile improvement or successful treatment for patients with PRD?
III) Are you aware of any models of “good services”? What is it that makes these good?
IV) What (if anything) do you think needs to be in place to care for these patients? Do you envisage any barriers to the implementation of these services?
