# Supplementary material for: A qualitative exploration of information access and provision for cancer survivors experiencing chronic bowel symptoms after pelvic radiotherapy: informing a framework to guide improvements to information provision
Source: Support Care Cancer. 2026 Sep 22;34(10):999. doi: 10.1007/s00520-026-11040-y (PMC13597550; doi:10.1007/s00520-026-11040-y)
Supplement: Supplementary file 2 [file 520_2026_11040_MOESM2_ESM.docx]

**Supplementary Table 1: Additional Verbatim Quotes**

| **Theme/ Topic** | **Verbatim Quote** | **Respondent** |
| --- | --- | --- |
| **Pre-Treatment** |  |  |
| *Provision* |  |  |
|  | *No, none whatsoever. No, I mean they were lovely who did the radiation people, lovely nurses, but not any of that information, no.* | ID101, female, 66 years |
|  | *The hospital gave me information at the start, but it wasn’t highlighted that erm…that there could be an ongoing problem caused by the radiotherapy.* | ID103, male, 66 years |
|  | *…they did explain the long-term effects, I was given lots of literature to read, and from MacMillan [cancer charity] their books are pretty good* | ID110, female, 48 years |
|  | *It’s explained in the small print isn’t it? Whatever they do to you is a small print kind of message, but some of the small print actually looms large in your life.* | ID113 female, 58 years |
|  | *I would say I had every bit of information I needed from the hospital, they were very, very good….*  *They spoke to me about it, erm give me leaflets about it. … everything I needed it was excellent.* | ID121, Male, 78 years |
|  | *our oncologists probably go through most of it, because they’ll go through a very detailed thing about the radiotherapy, dosage, side effects, all the rest of it. They will talk through short and long-term bowel dysfunction, etc.* | ID213, Colorectal Surgeon |
| *Reception* |  |  |
|  | *I was kind of gritting my teeth and thinking I’ve got to do this* (have radiotherapy) *and so I kind of merely flicked the booklet.* | ID114, female, 66 years |
|  | *you did not have space to think about that kind of thing.* | ID115, male 66 years |
|  | *I was given some* [information] *on the after-effects of radiotherapy. …it was a great heap of paper about this, that and the other and you flicked through it, and you read that 1 in 100 gets 6 side effects, or might get 1 of 6 side effects and 1 in 1000 might. And it doesn’t actually penetrate that which category you’re going to be in, and you don’t frankly take it very seriously…* | ID118, male 78 years |
|  | *I suspect the information that’s taken away is much more about what’s going to happen to them in the next few weeks rather than the next couple of years.* | ID212 Oncologist |
| *Use* |  |  |
|  | *I thoroughly researched the difference between having the two options, radiotherapy or removal* [prostatectomy]. | ID103, male, 66 years |
|  | *So, to me and I think probably the vast majority of people would say, I’m not just speaking for me, there’s not a choice in there is there, tell me what you like but I’m still going to have this treatment, there’s no way I’m going to turn round and say I’m not having this treatment.* | ID116, female, 51 years |
|  | *I think that whenever it was explained to me about the pelvic radiotherapy, I probably didn’t know enough about it to make an informed decision about the treatment. … I wasn’t maybe given enough information, couldn’t make that informed decision…* | ID117, female, 56 years, |
| **Recognising symptoms** | | |
|  | *when I was told it (the bleeding) was radiation proctitis, then I was able to research it more and fully understand it. … sometimes you might look in the pan and….it’s red …I’m not worried about it at all* | ID103, male, 66 years |
|  | *it would have been nice to have been told it may not last 12-18 months it may be forever. And you know to have a bit more information would have been nice.* | ID111, female, 65 years |
|  | *if they’d told me at the beginning this is likely, it could look like this, if you get this, this is more than likely to be radiation damage then would have been helpful.* | ID113 female, 58 years |
|  | *I was incontinent in the middle of my sleep. Nobody told me that you know. You might be able to do a bit of research, you would be aware of it, but it’s a shock when you have these incidences that happen.* | ID117, female, 56 years |
|  | *their worry is about the bleeding itself … it doesn’t seem to be something that’s really discussed prior to them having their radiotherapy treatment…* | ID206, Clinical Nurse Manager |
| **Managing Symptoms** | | |
|  | *if you want one severe complaint about another professional, it would be the dieticians, I don’t know how people can study for 3 or 4 years and all they do is give you a low SG sheet, that I gave out as a nurse 30 years ago … and they’re still giving out the same sheet …the difference in just giving ya a nutrition sheet, a diet sheet and somebody trying to translate that into how you can live that, that’s the difference to me in a dietician and nutritionist.* | ID106, female, 61 years |
|  | *…is there any more exercises I can do, is there anymore diet related things I can do?* | ID111, female, 65 years |
|  | *I found that really hard to get any information on that (diet). I’ve literally had to sort that all out myself.* | ID116, female, 51 years |
|  | *…maybe there needs to be more advice given to the patient in terms of diet.* | ID128, male, 71 years |
|  | *… they need some proper advice.* | ID203, Therapeutic Radiographer |
|  | *any diet restriction is hard…finances play a big role in what people can afford to buy and eating habits and likes and dislikes. … I give education and information… from …what they’ve told me they eat, that might be a risk and a problem, and I give them ideas of how to swap and change that. I give them resources to try and help support making choices that aren’t necessarily healthier, but they might be … practical suggestions that might be workable for them.* | ID207, Dietician |
| **Information seeking and sources** | | |
|  | *there is so much bad information on the web that to focus on specific erm recognised charities and cancer support groups is the way forward* | ID103, male, 66 years |
|  | *… I went back to the support group and said can you tell me in the group whose had these three different methods of treatment and so I spoke to guys that had had it done and what their side effects were.* |  |
|  | *MacMillan … all during the treatment you’re able to go in …lots of information booklets you can see read and people you can talk to about what you’re going through and er that was very helpful* |  |
|  | *I’ve literally had to fumble my way through, to find useful information and to finally get help and to be able to help other people.* | ID104, female, 51 years |
|  | *…it was only because the lady in the next bed said to me right this is what you need, you need to get yourself some barrier cream or nappy cream…* | ID110, female, 48 years |
|  | *the most helpful advice I think I have had is off people that have got it, that are further down the line than me, that have given me little tips you know of what to do, how to deal with stuff* | ID110, female, 48 years |
|  | *I do belong to a few groups on Facebook.* |  |
|  | *sometimes people set up a group online and they tell you the wrong information and they don’t necessarily like it when you correct them.* |  |
|  | **...***it was my hairdresser actually, she said to me have you seen the app called foodmap or fodmap…* | ID111, female, 65 years |
|  | *I did go on to a bowel cancer forum. And then to be honest I realised in a lot of respects how lucky I was, you know with my symptoms.* |  |
|  | *I don’t normally go to them,* (support groups) *but I have been going just a little bit recently to try and find some information out from others about basically who I can go to in* [UK geographic region] *who might be able to help* (with symptoms) | ID112, female, 62 years |
|  | *I’m someone who in some areas of life, likes to have information. With illness I suppose I find myself in a vulnerable situation* [and therefore want more information] | ID114, female 63 years |
|  | *I have access to all the medical literature that I want basically so I can read the original papers and the reviews on this kind of thing.* | ID115, male 64 years |
|  | *I know the quality of sources, where the information’s coming from, verification, peer review and I know where to get decent information from. I know the dangers of Google and Doctor Google.* |  |
|  | *I thought I’d actually sign up to that* (online PRD group)*, so that’s my first one tomorrow night….I’m looking to find out if other people are the same as me. Just to hear their experiences really.* |  |
|  | *I don’t like to be bothered …. I’m definitely not in the need to enquire …* | ID119, male, 79 years |
|  | *I couldn’t really see any benefit going down that road* (of joining attending a support group). | ID121, male, 78 years |
| **Challenges in information provision** | | |
| *Timing* |  |  |
|  | *… it was erm five/ten-minute meeting sat in a room just prior to your first appointment erm where your head space really is not taking a lot in* | ID104, female, 51 years |
|  | *…the problem isn’t the treatment, the problem isn’t my relationship or lack of it, with the consultant, for me it was a lack of information at a time I could have done with it.* | ID114, female, 63 years |
|  | *I don’t necessarily expect at the point of consent to be taken through every possible thing you know, in detail, every possible thing that could go wrong … but during the stage where I did have severe symptoms I would have welcomed maybe more of an explanation of what might be happening … in one of the calls with one of the doctors maybe* |  |
|  | *I think sometimes time has elapsed and so perhaps they need a little bit of reminder of that, if it’s some years down the line* | ID202, Nurse Endoscopist |
|  | *we’ve still got all those legacy patients out there from years ago that haven’t had that information.* | ID203, Therapeutic Radiographer |
|  | *Do you tell them at the beginning because they’re not listening because they’ve just been told they’ve got cancer and they don’t hear anything else, or do you tell them at the end of their treatment?* |  |
|  | *you just have to go back again. So, it’s about checking again later on down the line and don’t assume someone’s been given the information. It’s always good to repeat it. But… I’m a proponent of sooner the better.* | ID207, Dietician |
| *Format* |  |  |
|  | *…did I take it all in, when they gave you leaflets that you could read in your own time? I don’t know.* | ID101, female, 66 years |
|  | *The problem is as you all know, the consultant’s too busy to be able to sit down and spend half an hour with ya, telling you what’s the problems are going to be.* | ID103, male, 66 years |
|  | *I was given lots of literature to read, and from MacMillan* [cancer charity] *their books are pretty good* | ID110, female, 48 years |
|  | (information on late-effects might be given) *in one of the calls with one of the doctors maybe… I’m always conscious of not wanting to waste peoples’ time when there are others who need it. I’m often quite happy to well, this is what it could be and here’s something you could read about it, take this away and there’s this website you can look at. So… probably in person and then here’s where to find more information* | ID114, female, 63 years |
|  | *there’s a huge amount of information that’s being taken and delivered in a short consultation space* | ID208, Oncologist |
| *Content* |  |  |
|  | *it should be more about how do you have that…especially with employee, employers, and you know is there ….a way of explaining it to them, you know that this is a long-term thing, can flare up and all that kind of stuff…* | ID108, female, 34 years |
|  | *…it* (printed information) *goes from you being like a teenager and you know having all your family there to look after you and being young and it’s all colourful information … and then, the next bit is everything said, discuss this with your partner.* |  |
|  | *…there’s a lot of the medical side, but not so much of the personal side. I think with this disease that doesn’t get mentioned enough...* | ID110, female, 48 years |
|  | *I think the information has got better for patients and also for the medical profession as well* |  |
|  | *the pelvic radiation disease association … told me about a doctor that had been doing lots of research about it and into it and he’d come over a lot of information. But it just seemed to take a really long time to filter down to like, you know even the bowel doctors and even longer to get to a GP.* |  |
