# Supplementary material for: A qualitative exploration of information access and provision for cancer survivors experiencing chronic bowel symptoms after pelvic radiotherapy: informing a framework to guide improvements to information provision
Source: Support Care Cancer. 2026 Sep 22;34(10):999. doi: 10.1007/s00520-026-11040-y (PMC13597550; doi:10.1007/s00520-026-11040-y)
Supplement: Supplementary file 3 [file 520_2026_11040_MOESM3_ESM.docx]

**Supplementary Table 2: A Provisional Framework for Information Access to Support Patient-Centred Care and Supported Self-Management for Cancer Survivors Treated with Pelvic Radiation.**

| **Time relative to radiotherapy** | **Primary purpose of information** | **Likely information needs of survivors** | **Possible sources for information provision/ access** | **Issues and challenges for information provision** | **Practical considerations for information provision** |
| --- | --- | --- | --- | --- | --- |
| Pre-treatment | - Informed consent. - Shared decision-making. | - Awareness of possible late-effects, and risk (personalised if possible). - Treatment choices. - Information sources including trusted online sources, charities and support groups. | - Initially through health service (Therapeutic Radiographers & perhaps Cancer Nurse Specialists). - Subsequently also through charities and support groups. | - Primary concern is survival and immediate treatment. - Information may not be taken in or retained. - Need to avoid causing unnecessary worry as not all will experience late-effects. | - Inclusion of family / caregivers - Layering of information allowing progressive access to detail if desired - Add digital / multi-media channels |
| End of treatment | - Support patient-centred care. - Manage ongoing side-effects of radiotherapy. - Support self-monitoring. | - Likely and possible trajectories of ongoing symptoms and their management. - Awareness of possible late-effects. - Repeat information sources including trusted online sources, charities and support groups. | - Health service (perhaps verbally through Cancer Nurse Specialists plus printed material and digital material, perhaps personalised for access through smartphone). | - May not be experiencing symptoms and may not experience them for months/years/decades – or may never experience them. - Printed material may not be retained; digital provision may lead to exclusion/inequalities. | - Scheduled reminders / refreshers (e.g. 6 monthly, annually, biannually) - Discharge information to GP alerting to possible late-effects |
| Occurrence of symptoms of late-effects | - Facilitate re-entry to system. | - Interpreting symptoms. - Re-entering health system through primary care or oncology. - Reassurance about recurrence. | - Online – health services and charities. - Phone/ app for checking and monitoring symptoms. - Retained printed materials. - Support groups. | - Survivor may not recognise symptoms or associate these with radiotherapy. - Primary care may not recognise symptoms or associate these with radiotherapy. - Embarrassment and gratitude can inhibit care-seeking. - Survivor may have moved location or health service staff may have changed. - Not all survivors join support groups. - Printed material may not be retained; digital provision may lead to exclusion/inequalities. - Symptoms may cause fear of recurrence. | - Frame messages to counter embarrassment and gratitude. - Provide a single contact point/person to navigate appropriate re-entry and follow-up in the event of late-effects. |
| Symptoms diagnosed / living with symptoms | - Supported self-management (potentially improving outcomes and reducing use of health service resources). - Minimising unmet information needs and improving quality-of-life. | - Access appropriate support for symptoms (e.g. physiotherapy, dietician, community incontinence teams). - Support to identify appropriate diet changes. - Awareness of potential medication options - Support to identify most effective use of medication. - Support to comply with physiotherapy / exercise. - Practical support and advice (e.g. toilet access). - Learning from experience of others (access to peer support/ survivor groups. - Psychological/ emotional management techniques. - Support to manage employment and financial problems including accessing benefits | - Health service. - Charities. - Support groups. | - Survivors may not know what support and services exist and therefore may not know what to look for. - Survivors may lack resources to access services/support privately if not available through health service. | - Accelerate provision of late-effects service nationally. |
| **Cross-Cutting Issues.** | | | | | |
| Timing & retention of information – need for repetition of and continued access to information, use of multiple channels/media, access to progressive layers of detail. | | | | | |
| Equity & exclusion (social, digital, sensory, cognitive, linguistic) - need for multiple formats, versions and languages and awareness of patchy service provision nationally. | | | | | |
| Balance autonomy and support – information provision as an element of *supported* self-management. | | | | | |
| Coordination/integration across health service – needs to encompass oncology, primary care, late-effects and need to recognise the value of a single point of contact. | | | | | |
| Experiences are not necessarily a linear progression through stages/ steps. These might be repeated, skipped or experienced in a different order. | | | | | |
| Context is crucial - health services (in UK) lacking resources, including staff time. | | | | | |
| Incorporating survivor perspectives through co-creation and feedback is essential for further development and refinement. | | | | | |
